# On the Use of Olive Oil in Blood Vessel Suturing

**Published:** 1918-09

**Authors:** 


**On the Use of Olive Oil in Blood Vessel Suturing.** By Dr. J. Shelton Horsley, Richmond. *The Annals of Surgery*, April 1918.

It has been found that vaseline or other paraffin oil used to prevent adhesions of the intestines after abdominal operation have an intensely irritating effect on the normal peritoneum of the dog and that it is absorbed very slowly if at all. On the other hand olive oil is non-irritating and slowly absorbed but it prevents adhesions in only about 20 o/o of the cases.

With these facts in mind the writer has tried olive oil in blood vessel suturing in four experiments on two dogs. His results have led him to believe that the use of olive oil in such cases will be followed by a higher percentage of success than will the use of vaseline or paraffin oils.

The chief objection to the method is that the threads when wrapped around the buttons on the suture staff do not hold as well when boiled in olive oil as they do when boiled in vaseline. This may be overcome by smearing sterile vaseline on the buttons of the suture staff immediately before used, care being taken that the vaseline does not come in contact with the vascular endothelium.

